# Data-Driven Assessment of Asphalt Pavement Rutting and Cracking Using Multisource Road Data for Preventive Maintenance

**DOI:** 10.3390/ma19214612

**Published:** 2026-10-30

**Authors:** Qichao Huang, Xingxing Li, Shihui Xu, Can Cui

**Affiliations:** 1 Investment Company of China Railway Seventh Group Co., Ltd., Zhengzhou 450016, China; 2 School of Water Conservancy and Transportation, Zhengzhou University, Zhengzhou 450001, China

**Keywords:** asphalt pavement, pavement deterioration, rutting, cracking, negative binomial regression, preventive maintenance

## Abstract

**Highlights:**

- A distress-specific framework links multisource road data to rutting and cracking risks.
- Traffic volume and distress rate ratio increase rutting risk; grade helps explain crack counts.
- Predicted crack counts identify five sections for priority inspection and maintenance.

**Abstract:**

Rutting and cracking caused by traffic, environmental exposure, and existing damage threaten asphalt pavement serviceability. This study develops an interpretable, data-driven framework for deterioration-risk assessment and preventive maintenance using the Xinxiang section of National Highway G107, China. Multisource data on pavement condition, historical distress and maintenance, traffic operations, road geometry, and environmental conditions were integrated. Rutting severity and crack counts were modeled as the response variables for permanent deformation and cracking, respectively. Rutting severity was modeled using binary logit regression, while crack counts were analyzed using standard negative binomial and negative binomial conditional autoregressive models. Peak-hour traffic volume and pavement distress ratio were positively associated with higher rutting severity. For cracking, pavement distress ratio was positively associated with crack counts in the standard model, while grade became more influential after spatial dependence was considered. The standard negative binomial model achieved a mean absolute deviation (MAD) of 20.192 and a mean squared prediction error (MSPE) of 922.203, slightly outperforming the spatial model. Predicted crack counts identified five candidate sections requiring priority field inspection. The framework converts routine road data into section-level evidence for targeted preventive maintenance.

## 1. Introduction

During long-term service, asphalt pavements are continuously subjected to the coupled effects of traffic loading, environmental factors, material aging, and pre-existing structural damage. Consequently, their performance gradually deteriorates, manifesting as various forms of distress, including rutting, cracking, raveling, and surface damage [1]. Among these distresses, rutting primarily reflects the accumulation of permanent deformation under repeated vehicle loading [2], whereas cracking is closely associated with material aging, fatigue damage, temperature effects, and pavement structural condition [1,3]. As a growing proportion of highway infrastructure transitions from the construction stage to long-term operation and maintenance, the timely identification of pavement deterioration risks and the implementation of appropriate preventive maintenance measures before further distress progression have become critical to extending road service life and maintaining pavement serviceability [4].

Pavement performance prediction provides an essential basis for pavement management and preventive maintenance [4]. Existing prediction methods can be broadly categorized as empirical/statistical, mechanistic–empirical, stochastic/probabilistic, and data-driven models [1,4]. Empirical models generally use regression relationships or performance deterioration curves to characterize the statistical associations among traffic loading, pavement age, structural parameters, and pavement performance. These models are structurally simple, readily interpretable, and relatively undemanding in terms of data; however, their ability to capture complex nonlinear relationships is limited. Mechanistic–empirical methods further integrate pavement structural responses with empirical damage transfer functions and can be used to analyze the evolution of rutting, fatigue cracking, and roughness. Their application, however, generally requires detailed material, structural, and traffic inputs, together with regional calibration [5]. To account for the randomness and uncertainty inherent in pavement deterioration, probabilistic models, such as Markov and Bayesian models, have also been widely applied to pavement condition transitions and long-term performance analysis [6]. In recent years, with the continued accumulation of long-term pavement performance databases and road condition survey data, data-driven methods, including artificial neural networks, random forests, XGBoost, and long short-term memory networks, have been extensively used to predict the international roughness index, pavement condition index, and individual pavement distresses, demonstrating strong capabilities for nonlinear modeling [7,8,9]. At the distress-specific level, deep neural networks and time-series models have been employed to predict rutting progression [10,11], while machine-learning methods have been used to predict thermal and fatigue cracking [12,13]. Overall, pavement performance prediction has gradually evolved from performance estimation based on individual empirical relationships toward the modeling of complex nonlinear relationships, the integration of multisource information, and the simultaneous improvement of predictive accuracy and model interpretability.

Recent studies have extended this progression through reviews of empirical pavement-performance models [14], dynamic Bayesian forecasting [15], geospatial analysis [16], explainable LSTM models [17], and comparisons of machine-learning algorithms for pavement damage prediction [18]. Despite substantial progress in pavement performance prediction, several issues warrant further attention. First, some studies characterize pavement condition using overall, composite, or functional performance indicators, such as the international roughness index and present serviceability index [1,19]. Although these indicators facilitate network-level pavement evaluation, they do not necessarily represent the same dimensions of pavement performance [19]. Previous research has further demonstrated that, although the international roughness index is associated to some extent with rutting and cracking, it cannot fully substitute for the separate characterization of individual pavement distresses [20]. Other studies have directly predicted rutting or cracking [10,11,12,13], but generally focus on a single distress type. The differential responses of different pavement distresses under comparable in-service conditions therefore remain insufficiently investigated. Second, recent pavement prediction research has placed increasing emphasis on predictive accuracy. Although complex machine-learning and deep-learning models can effectively capture nonlinear relationships, model interpretability remains particularly valuable when real-world road management data are limited [7,9]. From the perspective of preventive maintenance, determining the likely severity of pavement distress alone is insufficient. It is also necessary to identify how traffic loading, road geometry, and existing pavement condition are related to the risks of different distress types, thereby explaining the causes of variations in pavement deterioration risk. Finally, the outputs of previous studies have generally focused on predicted performance indicators or model accuracy. Although long-term pavement data have increasingly been used to evaluate maintenance effectiveness and support decision-making [21], further research is needed to translate distress-model outputs into road-section-level risk information and to use this information to identify road units requiring priority inspection and early intervention.

Recent highway-reconstruction research has also linked surface distress surveys, structural testing, and internal-damage indicators to section-specific maintenance recommendations [22]. From the perspective of preventive maintenance, the value of distress prediction lies not only in improving the accuracy of pavement condition estimation, but also in transforming pavement inspection and road operation data into risk information that supports proactive maintenance [4,21]. Therefore, rather than solely pursuing increasingly complex prediction algorithms, an analytical framework that offers adequate interpretability, distinguishes among the responses of different distress types, and further identifies high-risk road sections is equally important for practical road maintenance. This is particularly relevant to road management settings where multiyear continuous monitoring data are unavailable. In such cases, making full use of routinely collected data—including pavement condition surveys, traffic operations, road geometry, and maintenance history [11,21,23,24]—to identify susceptibility to pavement deterioration can provide a relatively low-cost approach with clear engineering interpretability for selecting candidate road sections for preventive maintenance.

Recent maintenance-decision studies have combined condition assessment with fuzzy multi-attribute decision making, Analytic Hierarchy Process-Technique for Order Preference by Similarity to Ideal Solution (AHP-TOPSIS), image-based multi-objective optimization, maintenance-prioritization models, and life-cycle environmental assessment [25,26,27,28,29]. These approaches provide a broader decision context for the section-level screening framework used here. Established studies have also reviewed neural-network applications and empirical pavement-performance models [30,31], developed international roughness index prediction using regression, Artificial Neural Network (ANN), Group Method of Data Handling (GMDH), support vector regression, and random forests [32,33,34,35], and advanced multi-objective and system-level pavement-maintenance optimization [36,37,38].

Against this background, this study uses the Xinxiang section of National Highway G107, China as a case study and integrates multisource road service information, including road design and pavement condition survey data, historical distress and maintenance records, traffic operation data, road geometry, and environmental factors, to analyze asphalt pavement deterioration risk. Rutting and cracking are selected as two representative pavement deterioration responses. Based on the characteristics of the respective datasets, a binary logit model is developed for rutting status, and a negative binomial regression model is established for crack counts. A negative binomial conditional autoregressive model is further employed to examine the effects of spatial correlation on cracking. Rutting is characterized by the mean rut depth per kilometer, with the original dataset comprising 96 direction-specific observations from the two travel directions. Cracking is counted for individual 100 m road units to improve the spatial resolution for identifying localized distress risks.

This study makes three application-oriented contributions. First, historical distress and maintenance records are used to model crack counts and rutting severity directly, rather than relying only on aggregate pavement-performance indicators. The interpretable models quantify the direction, magnitude, and significance of the explanatory-variable effects, supporting distress-specific inspection and maintenance measures. Second, the standard negative binomial model is compared with a negative binomial conditional autoregressive (CAR) model to determine whether the explanatory-variable effects remain stable after spatial correlation between adjacent sections is considered. Third, the model estimates are translated into section-level risk information and a staged screening process for priority field verification and preventive maintenance.

## 2. Study Area, Data, and Variables

### 2.1. Data Sources

Distress on arterial highway pavements results from the combined effects of traffic loading, pavement condition, and roadway geometry. This study examines sections associated with the G107 reconstruction project in Xinxiang and compiles pavement-distress, traffic-operation, pavement-condition, and geometric data to identify factors affecting crack frequency and rutting severity and to screen high-risk sections [39].

**Figure 1:**
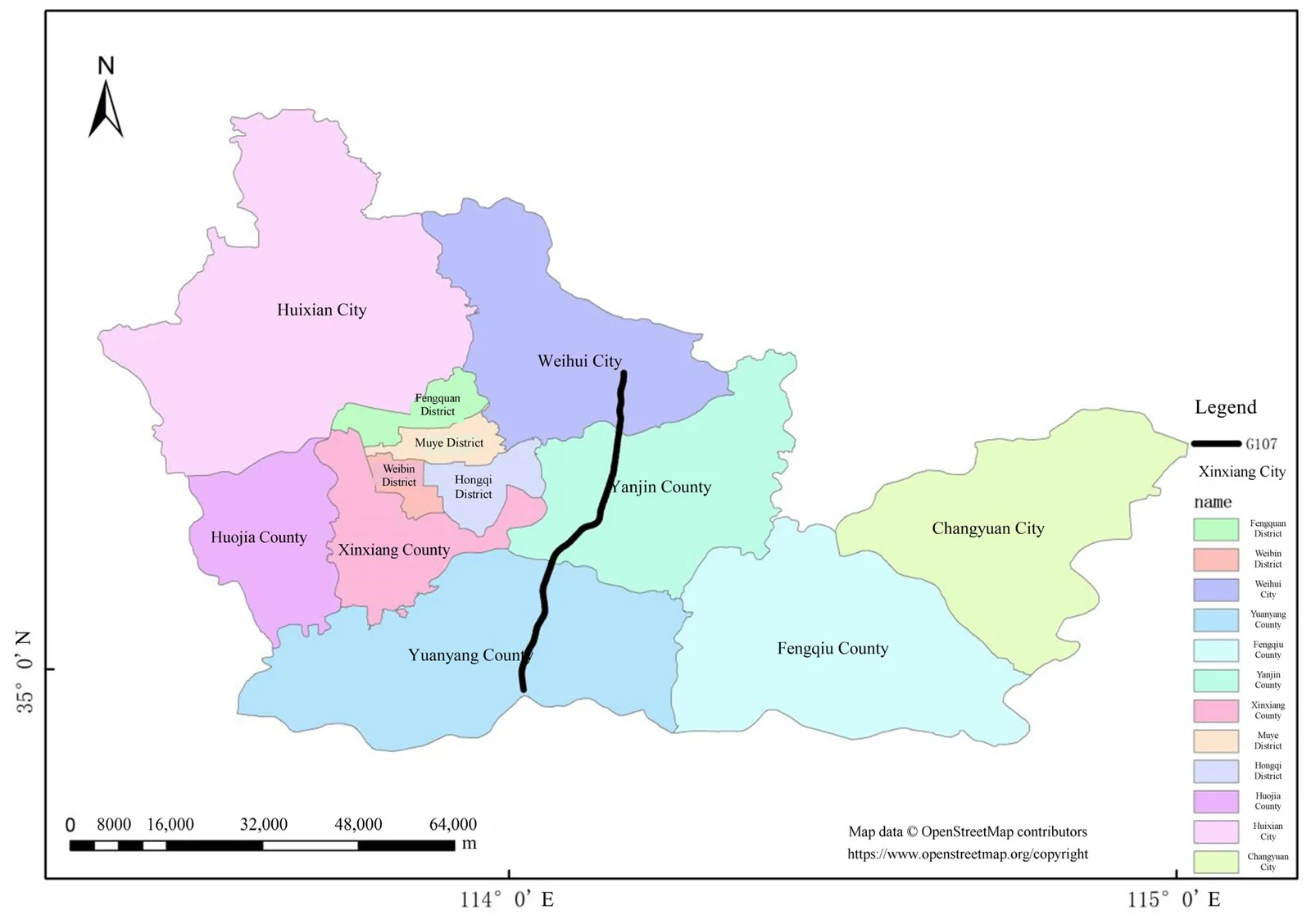
G107 Route Area Map.(The map was created by the authors using data from OpenStreetMap, available under the Open Database License (ODbL) [40]).

The G107 Xinxiang reconstruction project is part of the north–south first-class arterial highway network in Henan province, China. It passes through Weihui City, Yanjin County, and Yuanyang County and extends for 51.904 km, including 32.812 km of new roadway and 19.092 km of reconstructed roadway. The pavement is predominantly asphalt concrete. Pavement condition was surveyed from K4+000 to K51+904 over 47.904 km, with the main indicators measured in all traffic lanes. Figure 1 presents the G107 route area map. It was prepared by the authors using ArcGIS 10.2, based on administrative boundary data for Xinxiang and G107 route information obtained from OpenStreetMap [40].

Pavement-distress and condition data were obtained from engineering design documents, pavement-condition surveys, and roadway inspection and maintenance records collected from July 2023 to April 2024. Traffic data were collected at the intersections of G107 with S310, S311, and S227. Continuous observations from the Yanji West Interchange were used for S311, whereas 10 min classified video surveys were conducted at S310 and S227. Short-duration vehicle counts were converted to hourly traffic volumes. Truck proportion was calculated as the number of trucks divided by the total number of motor vehicles observed during the same survey period.

Roadway geometric data included section chainage and longitudinal grade. Absolute longitudinal grade was extracted from the route longitudinal profile and subgrade design tables and calculated as

(1) $$\left|i\right|=\left|\frac{\Delta H}{L}\right|\times 100\%$$

where Δ*H* is the design-elevation difference and *L* is the horizontal length. International roughness index (m/km) and skid resistance were obtained from the pavement-condition survey.

Pavement distress ratio represents the weighted distressed area as a proportion of the surveyed pavement area:

(2) $$\mathrm{DR}\;(\%)\;=\;100\;\times \;\frac{\sum_{i}\left(w_{i}A_{i}\right)}{A}$$

where *A* is the surveyed area, *A_i_* is the cumulative area of distress type *i*, and *w_i_* is its weight or conversion coefficient. In automated detection, *A_i_* was determined from 0.1 m × 0.1 m image grid cells containing the corresponding distress. Pavement distress ratio is reported in Table 1 and Table 2 as a decimal fraction.

### 2.2. Rutting Condition

A total of 96 observations of mean rut depth over 1 km sections were obtained for both travel directions. The 1 km scale was retained from the original pavement-condition dataset and reduces localized measurement fluctuations while matching common road-management units. The sample median of 4.08 mm was used as the statistical cutoff: observations below 4.08 mm were classified into the lower-rutting group (y = 0), whereas observations equal to or above 4.08 mm were classified into the higher-rutting group (y = 1). This sample-driven cutoff, rather than a technical-standard severity threshold, produced 47 lower-rutting observations (49%) and 49 higher-rutting observations (51%). Candidate explanatory variables included peak-hour traffic volume, truck proportion, skid resistance index, and pavement distress ratio. The descriptive statistics are summarized in Table 1.

### 2.3. Crack Counts

The study roadway was divided into 100 m analysis units, and the crack count in each unit was used as the response variable, resulting in 750 observations. This finer scale preserves localized crack clustering that could be smoothed by 1 km aggregation and provides sufficient observations for count and spatial analyses. Candidate explanatory variables included peak-hour traffic volume, truck proportion, international roughness index, absolute longitudinal grade, and pavement distress ratio. Crack counts are nonnegative integers and exhibited substantial dispersion. Table 2 summarizes the variables used in the crack count model.

### 2.4. Spatial Distribution of Pavement Distress

**Figure 2:**
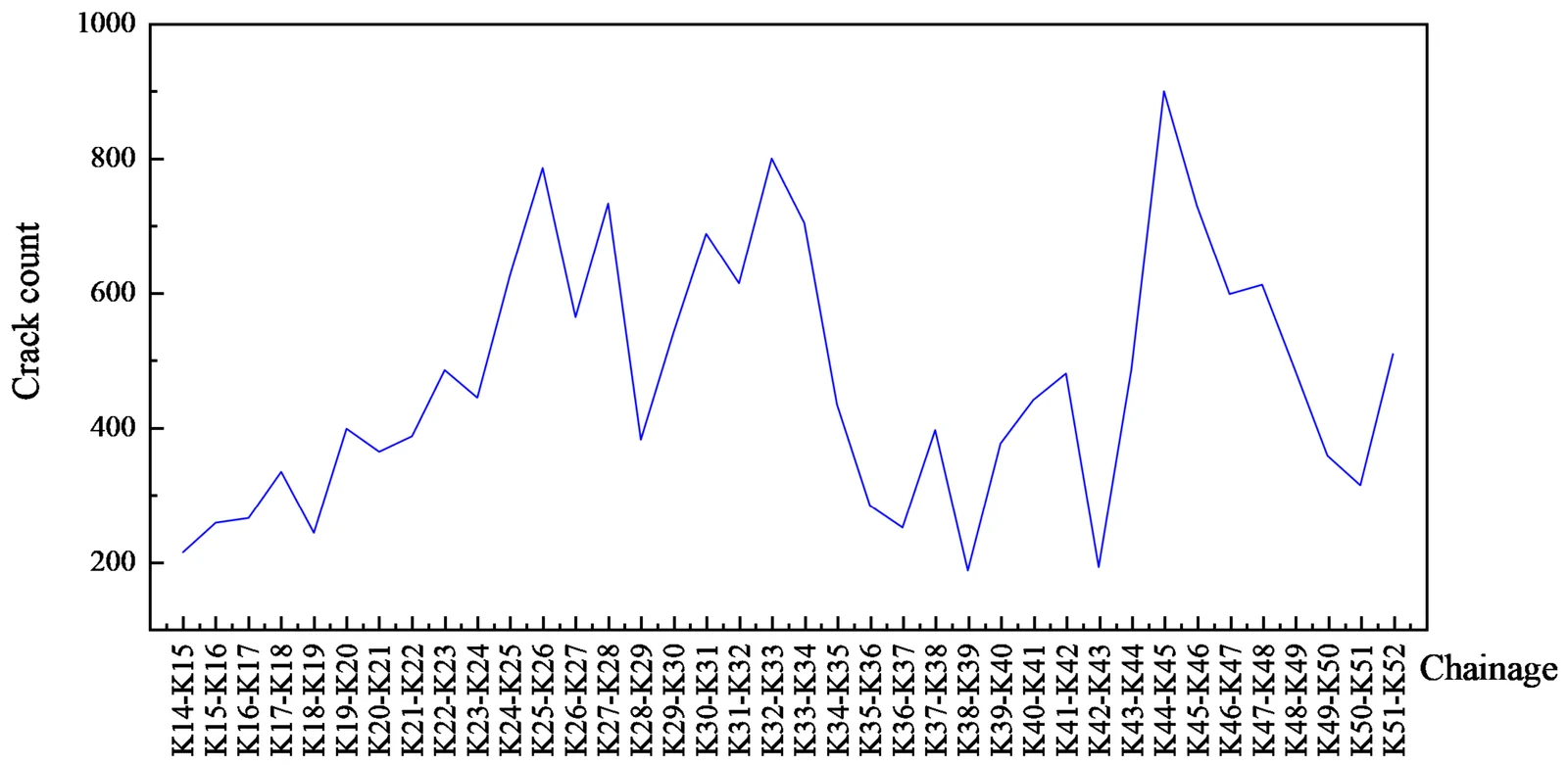
Spatial Distribution of Crack Counts.

A total of 750 segments were recorded in both travel directions. Crack counts varied markedly along the route and were concentrated primarily in K24-K28, K30-K34, and K45-K48. The rutting analysis included 96 km-level observations, with clear variation in mean rut depth among sections. The largest mean rut depth, 6.50 mm, occurred in K29-K30, whereas the smallest, 2.32 mm, occurred in K7-K8. Sections with relatively severe cracking and rutting partly overlapped sections with high pavement distress ratios, suggesting that existing pavement condition may influence the development of both distress types. This relationship is tested in the subsequent modeling analysis. The spatial distribution of crack counts along the study roadway is shown in Figure 2.

## 3. Methodology

### 3.1. Crack Count Models

Crack counts are nonnegative integers and were substantially overdispersed, so the Poisson equal-mean-and-variance assumption was inappropriate. The negative binomial model introduces a dispersion parameter and allows the conditional variance to exceed the conditional mean. The negative binomial specification also provides directly interpretable coefficients and incidence rate ratios. Let the crack count for section $Y_{i}$ follow a negative binomial distribution [41,42]:

(3) $$Y_{i}\sim \mathrm{Negbin}\left(\lambda_{i},\gamma \right)$$

where $\lambda_{i}$ is the expected crack count and γ is the dispersion parameter. Applying a log link to $\lambda_{i}$ yields the following linear predictor:

(4) $$\mathrm{In}\left(\lambda_{i}\right)\;=\;\beta_{0}\;+\;\beta_{1}\mathrm{In}\left({\mathrm{DVMT}}_{i}\right)\;+\;\sum_{k=2}^{p}\beta_{k}X_{\mathrm{ik}}\;+\;\varphi_{i}$$

where $X_{ik}$ is the value of the kth covariate for observation i, *p* denotes the total number of covariates included in the model, $\beta_{0}$, $\beta_{1}$, and $\beta_{k}$ are the coefficients to be estimated, and $\varphi_{i}$ is a spatially structured random effect for section i, accounting for residual spatial dependence. $\varphi_{i}$ follows the conditional autoregressive prior:

(5) $$\varphi_{i}\sim N\left(\frac{\sum_{j\neq i}w_{\mathrm{ij}}\varphi_{j}}{\sum_{j\neq i}w_{\mathrm{ij}}},\frac{1}{\tau_{c}\sum_{j\neq i}w_{\mathrm{ij}}}\right)$$

where $w_{ij}$ is an element of the spatial adjacency matrix W. If section i is adjacent to section j, then $w_{ij}=1$; otherwise, it equals 0. $\tau_{c}$ is the precision parameter (the inverse of the variance) [43,44].

The standard negative binomial model assumes conditionally independent section counts. However, adjacent 100 m sections may share pavement structure, loading, drainage, environmental exposure, and maintenance history. The CAR component was therefore used as a spatial robustness analysis to test whether the direction and significance of the explanatory-variable effects remained stable after residual spatial correlation was considered. An adjacency-based CAR structure was selected because the analysis units are contiguous road sections and can be integrated directly with the negative binomial count distribution.

For Bayesian estimation, noninformative prior distributions were assigned to the model parameters:

(6) $$\beta_{k}\sim N\left(0,\;1000\right)$$

(7) $$\frac{1}{\gamma }\sim \mathrm{gamma}\left(0.01,\;0.01\right)$$

(8) $$\tau_{c}\sim \mathrm{gamma}\left(0.01,\;0.01\right)\;$$

The model parameters were estimated using WinBUGS version 1.4.3 (MRC Biostatistics Unit, Cambridge, UK).

### 3.2. Rutting Severity Model

Rutting severity was classified into two categories and modeled using a binary logit model. Let *Y_i_* = 1 denote the higher-rutting group and *Y_i_* = 0 denote the lower-rutting group. The objective was to estimate the probability of higher rutting for section-level screening, and the logit model provides directly interpretable odds ratios. Linear regression is unsuitable for a binary response. A Probit model was also plausible, but the logit specification was selected because its odds ratios have more direct engineering interpretation. The probability of a higher-rutting observation is:

(9) $$P(Y_{i}=1|X_{i})=\frac{\exp(\beta_{0}\;+{\;\beta }^{T}X_{i})}{1+\exp(\beta_{0}\;+{\;\beta }^{T}X_{i})}$$

where where P denotes probability, *X_i_* is the vector of explanatory variables and beta is the vector of parameters to be estimated, and the superscript denotes matrix transposition. A positive coefficient indicates that an increase in the corresponding explanatory variable raises the probability of higher rutting. Parameters were estimated by maximum likelihood, and overall model fit was evaluated using the likelihood-ratio test and McFadden’s pseudo-R-squared [45,46].

### 3.3. Data Screening and Model Comparison

The negative binomial and spatial models were compared using mean absolute deviation (*MAD*) and mean squared prediction error (*MSPE*), with smaller values indicating lower in-sample prediction error [47,48].

(10) $$MAD=\frac{1}{n}\sum_{i=1}^{n}\left|Y-Y_{i}\right|$$

(11) $$MSPE=\frac{1}{n}\sum_{i=1}^{n}{\left(Y-Y_{i}\right)}^{2}$$

## 4. Results

### 4.1. Crack Count Model Results

#### 4.1.1. Model Comparison

Before modeling, correlation coefficients were used to assess preliminary relationships between each explanatory variable and the response, and variance inflation factors (VIFs) were used to examine multicollinearity. The largest VIF was 1.23 for the crack-count model and 1.87 for the rutting severity model, both below 5. Table 3 compares the standard negative binomial model with the negative binomial CAR model. The standard model yielded MAD and MSPE values of 20.192 and 922.203, respectively, compared with 20.240 and 939.696 for the CAR model. Although both error metrics were slightly lower for the standard model, the differences were only 0.23% for MAD and 1.86% for MSPE, indicating similar in-sample predictive performance. The standard negative binomial model was therefore used to interpret the effects of the explanatory variables, whereas the CAR model was used to determine whether the direction and statistical significance of these effects changed after spatial dependence between adjacent sections was considered.

#### 4.1.2. Negative Binomial Model Results

Table 4 presents the parameter estimates for the standard negative binomial model. The model was significant overall, indicating that the selected covariates jointly explained variation in crack counts over 100 m sections. Pavement distress ratio was positively associated with crack count, with a coefficient of 2.642. A 0.10 increase in pavement distress ratio was associated with a 30.2% increase in the expected crack count. Existing cracking, potholes, and raveling can reduce structural continuity and waterproofing. Moisture entering the surface or base layers may weaken asphalt-aggregate bonding and accelerate crack propagation under temperature variation and repeated vehicle loading [39,49].

Absolute longitudinal grade had a coefficient of 0.226 (*p* = 0.072). It provided exploratory evidence of a positive association at the 10% level. The corresponding international roughness index was 1.254, suggesting that steeper grades were associated with higher expected crack counts. Changes in longitudinal grade affect vehicle traction, braking, runoff, and internal moisture movement, which may accelerate cracking where drainage is inadequate [39].

Truck proportion had a negative coefficient (*p* = 0.094) and was significant only at the exploratory 10% level. Peak-hour traffic volume and international roughness index had *p*-values of 0.449 and 0.622, respectively, and neither effect was statistically significant. Under the present sample and model specification, the data therefore provide insufficient evidence that either variable independently affects crack counts.

#### 4.1.3. Negative Binomial CAR Model

Compared with the standard negative binomial model, the CAR model explicitly accounted for spatial dependence between adjacent road sections. In the standard model, pavement distress ratio had a significant positive effect, whereas absolute longitudinal grade provided only exploratory evidence. In the CAR model, absolute longitudinal grade had a significant positive effect, whereas pavement distress ratio was not significant at the 90% credible level. These differences indicate that the estimated effects are sensitive to the treatment of spatial dependence. Table 5 presents the posterior estimates of the negative binomial CAR model.

### 4.2. Rutting Severity Model Results

At the 0.05 significance level, the likelihood-ratio test yielded χ^2^(4) = 55.02 and *p* < 0.001, indicating that the full model provided a significantly better fit than the intercept-only model. In addition, the McFadden pseudo-R^2^ value of 0.4135 indicated that the binary logit model achieved a comparatively good fit. Table 6 presents the parameter estimates, which are interpreted below in terms of statistical significance and effect direction.

Peak-hour traffic volume had a coefficient of 0.011 (*p* = 0.005) and was positively associated with rutting severity. Higher traffic volumes concentrate vehicle loading within shorter periods, causing repeated tire loading in the same wheel paths and accelerating densification and shear deformation of the asphalt mixture [39,49].

Pavement distress ratio had a coefficient of 23.745 (*p* = 0.004) and was positively associated with rutting severity. For each 0.01 increase in pavement distress ratio, the odds of higher rutting increased by approximately 26.8%; the 95% confidence interval for the increase ranged from 7.7% to 49.2%. Cracking, potholes, and localized raveling reduce structural continuity and load-bearing capacity, making weak locations more susceptible to nonuniform stress and wheel-path densification and shear deformation. Rutting may also promote surface ponding and concentrate wheel loads, further aggravating pavement damage [39,50].

Truck proportion had a coefficient of −13.091 (*p* = 0.046). A one-percentage-point increase in truck proportion corresponded to a 12.3% decrease in the point estimate of the odds of higher rutting, with a 95% confidence interval for the odds ratio of [0.772, 0.997]. Although significant at the 5% level, the upper confidence limit was close to 1, indicating borderline statistical evidence. The result may reflect the small number of traffic survey sites, short-duration observation error, and imprecise matching of traffic data to kilometer sections. Sections with high truck proportions may also have lower total traffic volumes, lower operating speeds, or greater maintenance inputs, causing truck proportion to capture unobserved section characteristics.

Skid resistance had a coefficient of −0.012 (*p* = 0.831), an odds ratio of 0.988, and a 95% confidence interval of [0.883, 1.104]. It was not significantly associated with rutting severity and should not be used as a primary screening indicator for rutting risk on the study roadway.

## 5. Discussion and Preventive Maintenance Implications

Distress frequency and severity describe different aspects of pavement condition. Unlike network-level studies centered on functional or composite indicators such as international roughness index and pavement condition index, this study directly models crack counts over 100 m sections and kilometer-level rutting severity. The explanatory variables themselves are established; the advance is to quantify their independent, distress-specific effects and test their sensitivity to spatial model specification. This complements established and recent prediction research [14,15,16,17,18,30,31,32,33,34,35].

Pavement distress ratio was a common factor in the two distress models. The standard negative binomial model and the binary logit model showed that higher pavement distress ratio values were associated with more cracks and a higher probability of severe rutting, respectively. The indicator therefore summarizes existing pavement damage and can support preliminary risk screening. In the negative binomial CAR model, however, pavement distress ratio had a positive effect only at the 90% credible level, indicating sensitivity to spatial dependence in the crack-frequency model. Screening based on pavement distress ratio should therefore consider continuity across adjacent sections rather than identify maintenance priorities from a single 100 m unit.

The models also identified distress-specific factors. Absolute longitudinal grade had a stable positive association with crack counts in the negative binomial CAR model, whereas peak-hour traffic volume had a significant positive effect in the rutting-severity model. Truck proportion was only marginally significant for rutting; its direction conflicted with conventional engineering expectations, and it may have been affected by limited survey sites, short-duration measurement error, and spatial matching error. Skid resistance was not significant. These results show that established variables do not necessarily retain independent explanatory power for every distress type.

Crack-frequency screening should focus on pavement distress ratio, absolute longitudinal grade, and distress continuity across adjacent sections, whereas rutting-severity screening should emphasize peak-hour traffic volume and pavement distress ratio. A practical workflow can use pavement distress ratio for initial screening, distress-specific factors for secondary review, adjacent-section continuity for spatial confirmation, and field inspection for final validation.

This workflow is a preliminary screening tool that complements existing pavement management and field inspection. It has not yet been shown to outperform current practice or alternative models because independent field verification, maintenance outcomes, and comparative cost data were unavailable. Its decision value lies in converting routine data into transparent section-level priorities for subsequent verification.

Comprehensive maintenance frameworks additionally consider structural testing, treatment alternatives, agency costs, environmental impacts, and system-level budget constraints [22,25,26,27,28,29,36,37,38]. The screened sections should therefore undergo field and structural evaluation before a final treatment is selected.

### Priority Road Sections and Preventive Treatments

**Figure 3:**
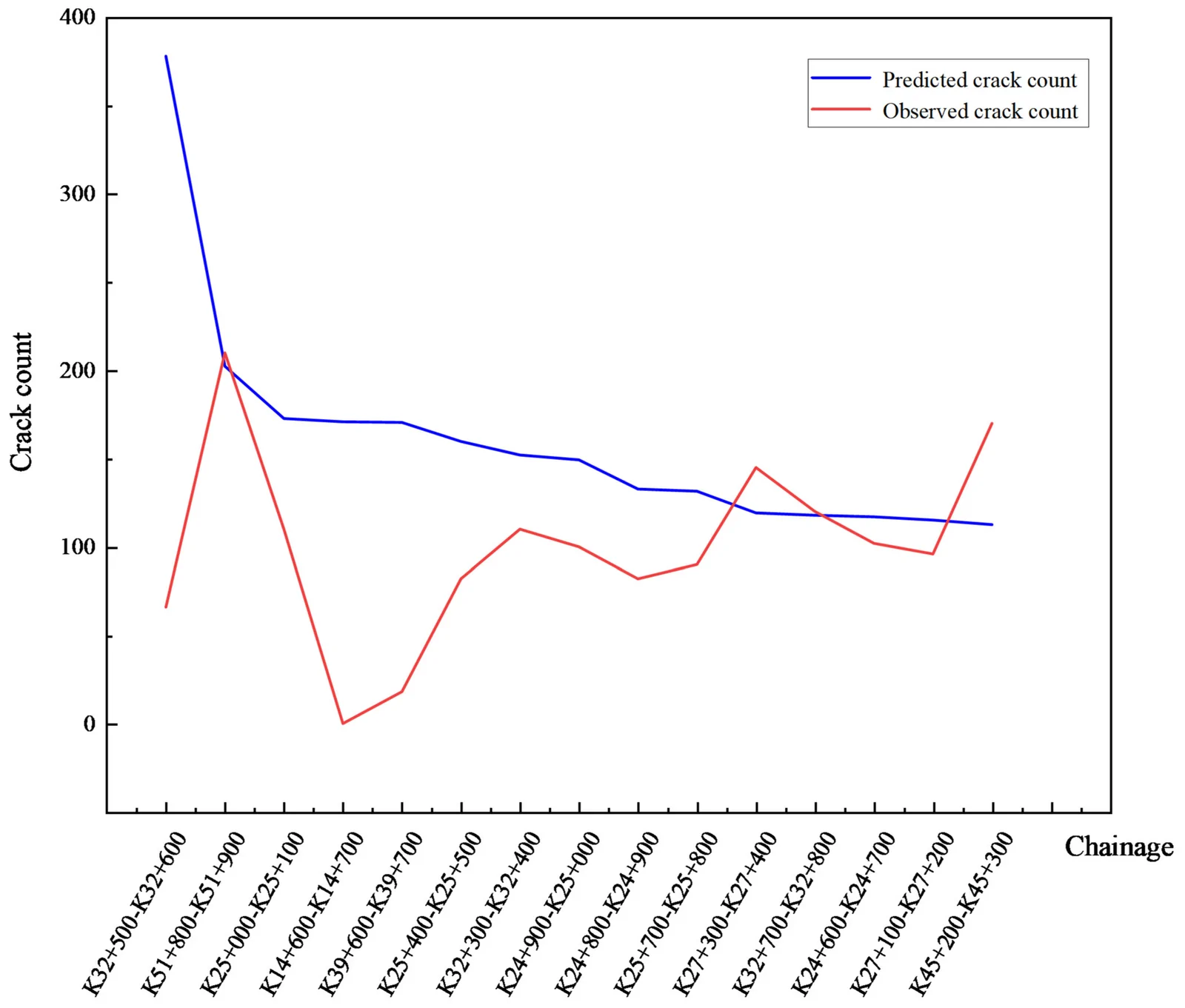
Observed and Predicted Crack Counts.

The negative binomial model identified K32+500-K32+600, K51+800-K51+900, K25+000-K25+100, K14+600-K14+700, and K39+600-K39+700 as candidate sections with the largest expected crack counts: 378.18, 202.40, 172.79, 170.85, and 170.53, respectively. Figure 3 compares the observed and predicted crack counts for these candidate sections. They require priority field verification before maintenance decisions are made, with attention to crack width, propagation direction, base-layer condition, and drainage.

Sections with numerous cracks but no evident structural failure may be treated by crack sealing, slurry sealing, or microsurfacing to limit moisture ingress and retard crack propagation [51,52]. If cracking is associated with base or subgrade damage, the weak structural area should be repaired before a surface seal is applied. Rutting on sections with high traffic volumes and high pavement distress ratio values warrants shorter rut-depth inspection intervals. Localized minor rutting may be corrected with surface treatments, whereas severe rutting may require milling and resurfacing, structural strengthening, or a rut-resistant asphalt mixture, depending on the underlying cause. The timing of preventive maintenance should reflect distress severity, progression rate, and traffic effects rather than a single inspection measurement [53,54].

## 6. Conclusions

This study developed a distress-specific, data-driven framework for assessing pavement deterioration risk and supporting preventive maintenance decisions on the Xinxiang section of National Highway G107. Multi-source data describing pavement condition, traffic operations, and roadway geometry were integrated at spatial scales appropriate for the two distress types. Crack counts in 100 m sections were analyzed using standard negative binomial and negative binomial conditional autoregressive models, whereas rutting severity in 1 km sections was examined using a binary logit model. The model estimates were further translated into section-level risk information for maintenance screening.

The standard negative binomial model achieved MAD and MSPE values of 20.192 and 922.203, respectively, and exhibited slightly better in-sample predictive performance than the spatial model. Pavement distress ratio was positively associated with crack counts in the standard model, while absolute longitudinal grade became the more prominent factor after spatial dependence between adjacent sections was incorporated. These results indicate that the estimated effects of pavement distress ratio and absolute longitudinal grade are sensitive to spatial model specification and that crack-risk screening should consider both local pavement condition and the continuity of deterioration across adjacent sections. For rutting severity, peak-hour traffic volume and pavement distress ratio were significantly and positively associated with the probability of belonging to the higher-rutting category.

From a maintenance perspective, pavement distress ratio can serve as a common preliminary screening indicator, while absolute longitudinal grade and peak-hour traffic volume provide distress-specific information for cracking and rutting, respectively. Five candidate sections requiring priority field verification were identified at K32+500-K32+600, K51+800-K51+900, K25+000-K25+100, K14+600-K14+700, and K39+600-K39+700.

Complete and consistent pavement-layer configurations, layer thicknesses, weigh-in-motion data, axle-load spectra, and cumulative equivalent standard axle loads were unavailable. Peak-hour traffic volume and truck proportion therefore represent only traffic exposure and vehicle composition. The candidate sections also lack independent field verification, and the framework has not been compared directly with existing inspection practice or alternative models.

Future studies should incorporate as-built pavement structure, continuous axle-load and multiyear condition data, maintenance costs, and post-treatment performance. Zero-inflated, hurdle, Bayesian spatiotemporal, continuous or ordinal rutting, and interpretable machine-learning models should be compared using out-of-sample error, classification accuracy, recall, calibration, field-verification hit rate, inspection workload, and cost. These extensions will test whether the framework provides measurable improvements in maintenance prioritization.

## Figures and Tables

**Table 1:** Descriptive Statistics of Variables Used in the Rutting Severity Model.

Variable	Mean	Standard Deviation	Maximum	Minimum
Peak-hour traffic volume (veh/h)	684.50	91.57	856	618
Truck proportion	0.590	0.048	0.644	0.535
Skid resistance index	89.39	6.39	98.25	66.23
Pavement distress ratio	0.075	0.053	0.226	0

**Table 2:** Descriptive Statistics of Variables Used in the Crack Count Model.

Variable	Mean	Standard Deviation	Maximum	Minimum
Peak-hour traffic volume (veh/h)	700.512	94.69	856	618
Truck proportion (%)	0.580	0.044	0.644	0.535
International roughness index (m/km)	2.73	1.57	9.88	0.85
Absolute longitudinal grade (%)	0.48	0.65	2.67	0.01
Pavement distress ratio	0.094	0.066	0.54	0

**Table 3:** Goodness-of-Fit Metrics for the Crack Count Models.

Model	MAD	MSPE
Negative binomial	20.192	922.203
Negative binomial CAR	20.240	939.696

**Table 4:** Parameter Estimates of the Standard Negative Binomial Crack Count Model.

Crack Count	Coefficient	Standard Error	*z*-Statistic	*p*-Value
Peak-hour traffic volume	−0.061	0.081	−0.76	0.449
Truck proportion	−0.127	0.076	−1.68	0.094
International roughness index	0.088	0.178	0.49	0.622
Absolute longitudinal grade	0.226	0.125	1.80	0.072
Pavement distress ratio	2.642	0.264	9.99	0.000
Prob > chi2	Model *p* < 0.001			

**Table 5:** Posterior Estimates of the Negative Binomial CAR Model.

Covariate	Coefficient	Standard Deviation	2.5%	97.5%
Peak-hour traffic volume	−0.026	0.036	−0.096	0.045
Truck proportion	−0.126	0.076	−0.271	0.026
International roughness index	0.096	0.177	−0.253	0.449
Absolute longitudinal grade	2.659	0.259	2.163	3.178
Pavement distress ratio	0.231	0.127	−0.011	0.484
Intercept	3.225	0.098	3.063	3.447

**Table 6:** Parameter Estimates of the Binary Logit Model for Rutting Severity.

Rutting Category	Coefficient	Standard Error	*z*-Statistic	*p*-Value	95% Confidence Interval
Peak-hour traffic volume	0.011	0.004	2.84	0.005	[0.003, 0.019]
Truck proportion	−13.091	6.545	−2.00	0.046	[−25.920, −0.261]
Skid resistance	−0.012	0.057	−0.21	0.831	[−0.124, 0.099]
Pavement distress ratio	23.745	8.313	2.86	0.004	[7.452, 40.039]
Prob > chi2	0.0000				

## Data Availability

The data presented in this study are available from the corresponding author upon reasonable request; they are not publicly available because the project-specific road inspection, traffic operation, and maintenance data are subject to confidentiality restrictions imposed by the data provider.
